# Low-Dose Aspirin for Prevention of Cardiovascular Disease in Patients with Chronic Kidney Disease

**DOI:** 10.1371/journal.pone.0104179

**Published:** 2014-08-05

**Authors:** Ae Jin Kim, Hye Jin Lim, Han Ro, Kwang-Pil Ko, Song Yi Han, Jae Hyun Chang, Hyun Hee Lee, Wookyung Chung, Ji Yong Jung

**Affiliations:** 1 Division of Nephrology, Department of Internal Medicine, Gachon University Gil Medical Center, Incheon, Korea; 2 Gachon University School of Medicine, Incheon, Korea; 3 Department of Preventive Medicine, Gachon University School of Medicine, Incheon, Korea; UNIFESP Federal University of São Paulo, Brazil

## Abstract

**Background:**

Chronic kidney disease (CKD) is a major risk factor for the development of cardiovascular disease (CVD). Previous trials have investigated the effects of low-dose aspirin on CVD prevention in patients with diabetes; however, patients with CKD were not examined. The role of aspirin in diabetics is controversial, and the available literature is contradictory. Therefore, we studied whether low-dose aspirin would be beneficial for patients with CKD, a group that is at high risk for CVD.

**Method:**

From a total of 25340 patients with CKD, 1884 recipients of low-dose aspirin (100 mg/day) were paired 1∶1 with non-recipients for analysis using propensity score matching. The primary endpoint was the development of atherosclerotic CVD, including coronary arterial disease, stroke, and peripheral arterial disease. Secondary endpoints included death from any cause, bleeding events, doubling of serum creatinine, and renal death.

**Results:**

The incidence of a primary endpoint of any atherosclerotic CVD was significantly higher in the aspirin users than in the non-users (*P*<0.001). Secondary endpoints, including all-cause mortality and composite bleeding events, were not significantly different between the aspirin users and the non-users. However, the doubling of serum creatinine levels (*P* = 0.001) and renal death (*P* = 0.042) were significantly associated with the use of aspirin.

**Conclusion:**

These results suggest that the use of low-dose aspirin in patients with CKD may have harmful consequences related to the development of CVD and renal progression.

## Introduction

Compared with the general population, individuals with chronic kidney disease (CKD) are at an increased risk for cardiovascular disease (CVD) and death from cardiovascular events [1]. CVD is the leading cause of mortality in individuals with varying degrees of CKD. Therefore, preventive measures for CVD are of great importance in patients with CKD. Previous studies have demonstrated that efforts to lower blood pressure [2], [3] and lipid levels [4] are effective for reducing the risk of CVD in patients with CKD. However, the importance of other potential preventive therapies, such as antiplatelet agents, remains controversial.

Aspirin has been shown to be effective in reducing cardiovascular morbidity and mortality in high-risk patients who have experienced myocardial infarction (MI) or stroke [5] and is recommended as a secondary prevention strategy for individuals with multiple risk factors such as hypertension, dyslipidemia, obesity, diabetes, and a family history of ischemic heart disease [6], [7]. The American Diabetes Association and the American Heart Association recommend low-dose aspirin (75–162 mg) for adults with diabetes who have no previous history of vascular disease, a 10-year risk of CVD events that is greater than 10%, and no increased risk of bleeding [6].

Several potential mechanisms have been proposed for the increased risk of CVD in patients with CKD, including increased oxidative stress [8], inflammation [8], platelet dysfunction [9], accelerated atherosclerosis [10], and attenuated response to antiplatelet agents [11]. Similar to diabetes, CKD has recently been considered to be equivalent to coronary artery disease [12], [13]. Although antiplatelet agents have been shown to reduce the risk for major CVD events in patients with coronary artery disease and in those with an equivalent disease such as diabetes, limited data exist regarding the use of antiplatelet agents in patients with CKD. This is largely because these patients were systematically excluded from large randomized trials [14]. Additionally, there is concern over whether chronic aspirin use can potentially lead to an increased risk of hemorrhage after treatment with an antiplatelet agent, as patients with CKD have abnormal platelet function [15]. There is substantial uncertainty regarding the risk/benefit balance associated with the use of antiplatelet agents by CKD patients. Accordingly, the purpose of this study was to evaluate the effects and safety of low-dose aspirin used for the prevention of CVD in patients with CKD, a group that is at high risk for CVD.

## Materials and Methods

### Ethics statement

This investigation was approved by the institutional review board (IRB) in Gachon University Gil Medical Center and was in accordance with the principle of the Helsinki Declaration II (GCIRB2938-2012). This study was a retrospective observational design and did not include any interventions, therefore written informed consent was waived after confirmation of IRB.

### Study design and setting

This study was a retrospective Propensity score (PS)-matched analysis of the effect of low-dose aspirin therapy on the development of CVD.

### Participants

**Figure 1:**
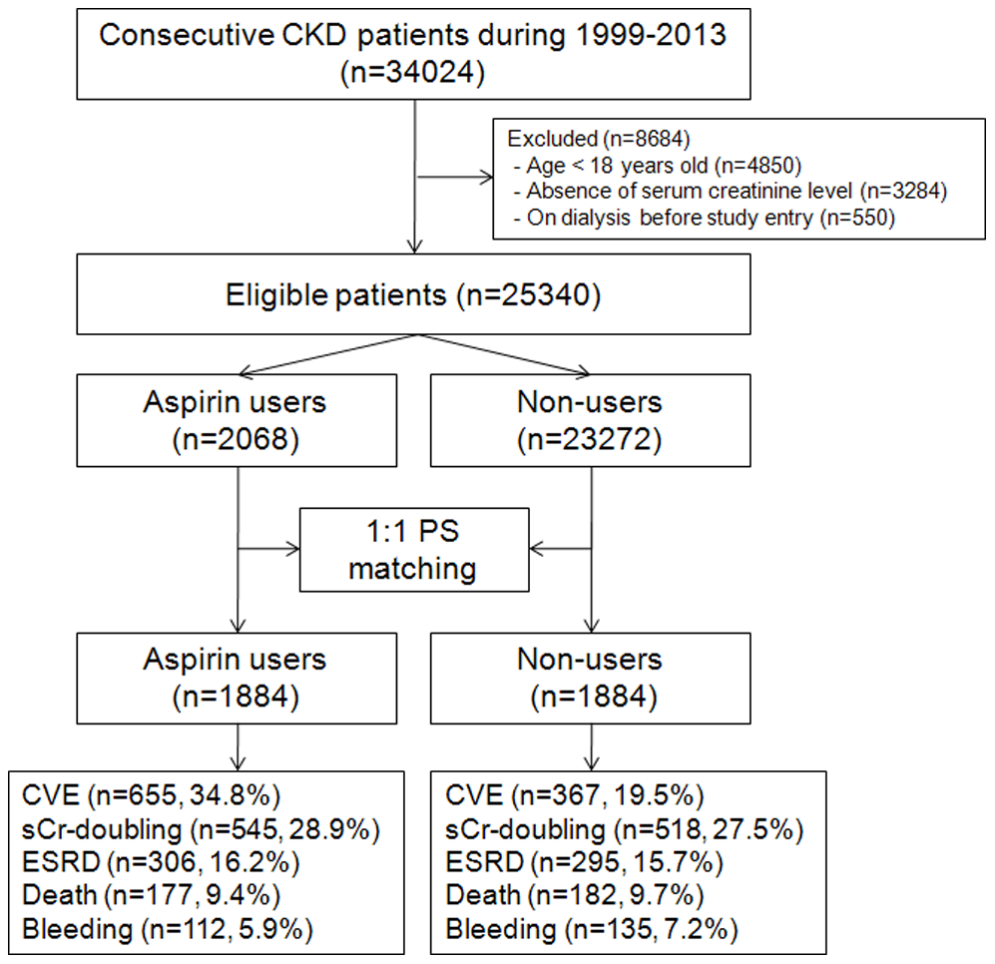
Cohort formation.

Patients who presented to Gachon University Gil Medical Center, which is a tertiary teaching hospital, with renal problems between November 1, 1999 and June 30, 2013 were screened for inclusion in this study. A total of 34024 patients with CKD were identified. The following groups of patients were excluded: (i) patients who were under 18 years of age (n = 4850); (ii) patients whose medical records were lacking information on serum creatinine levels (n = 3284); and (iii) patients who received dialysis prior to entry in the study (n = 550). Therefore, 25340 patients were included in the final analysis (Figure 1). The Institutional Review Board of our hospital approved the research protocol and methods.

### Variables

The patients’ demographic and clinical and laboratory data were obtained from electronic medical records. We obtained the following demographic data: age, gender, Body mass index (BMI), comorbidities (diabetes, hypertension, history of CVD, and history of peptic ulcer disease). Other comorbid conditions, such as angina pectoris, myocardial infarction, other ischemic heart disease, atrial fibrillation, heart failure, hypertension, and cerebrovascular disease, were identified using the validated *ICD-10* codes. Cardiovascular disease (CVD) was defined as angina pectoris, myocardial infarction, other ischemic heart disease, atrial fibrillation, heart failure, or cerebrovascular disease. Medications included renin-angiotensin-aldosterone system (RAAS) blockers (angiotensin-converting enzyme inhibitors and angiotensin receptor blockers), statins, beta-blockers, calcium channel blockers (CCBs), diuretics, warfarin, clopidogrel and cilostazol. The laboratory data included levels of proteinuria as measured by a dipstick test, serum creatinine levels, and the levels of hemoglobin, albumin, cholesterol, triglycerides, high-density lipoprotein (HDL)-cholesterol, calcium, phosphorus, and uric acid at the time of the initial hospital visit. We estimated the glomerular filtration rate (eGFR) using the following 4-variable Modification of Diet in Renal Disease (MDRD) Study equation. CKD was defined by a specific value of the eGFR (<60 ml/min/1.73 m^2^), the presence of proteinuria (defined as trace or greater by dipstick), or both on at least 2 occasions ≥3 months apart [16].

### Outcome end points

The primary end point was the development of any atherosclerotic event, which was a composite of significant coronary artery disease that required angioplasty (including ballooning and stenting), ischemic stroke, and peripheral vascular disease. Adverse events that were defined as composite bleeding included gastrointestinal bleeding, hemorrhagic stroke, and hemoptysis. Other secondary endpoints included death from any cause, time to doubling of serum creatinine, and renal failure that was defined by the occurrence of end-stage renal disease (ESRD) that required renal replacement therapy. Comparisons of outcome end points were performed on the basis of time until the first event according to the intention-to-treat principle, and they included all patients in the groups to which they were assigned (follow-up was censored on the day of the last visit).

### Statistical analysis

Because the patients in this study were not randomly assigned to an aspirin treatment group, we used the PS to reduce any potential confounding and selection biases. Two different types of analyses in assessing the association of aspirin with outcomes were used; intention to treat (ITT) for the unmatched cohort and PS for the matched cohort. In addition, to reduce potential confounding and treatment selection bias, we performed rigorous adjustment for significant differences in baseline covariates with the use of PS matching. We calculated the PS for each patient by modeling the probability of receiving aspirin. A multivariable logistic regression analysis model was generated to predict the probability of receiving aspirin, given the following set of covariates: age, gender, BMI, diabetes, hypertension, history of CVD and peptic ulcer disease, proteinuria, baseline eGFR, baseline levels of hemoglobin, white blood cells, platelets, albumin, cholesterol, triglycerides, HDL-cholesterol, calcium, phosphorus, and uric acid in addition to the use of RAAS blockers, statins, beta-blockers, CCBs, diuretics, warfarin, clopidogrel and cilostazol. Using these covariates, a PS was calculated for each patient. We subsequently used the derived PS values to match 2068 aspirin users with non-users at a ratio of 1∶1 using the Greedy matching algorithm (8 to 1 digit match). After all PS matches were performed, we assessed the balance in baseline covariates using the standardized mean difference, paired t-test, and McNemar’s tests, as appropriate, for continuous and categorical variables (Table 1). We analyzed all available data without the imputation of missing values. PS matching was performed with the Statistical Analysis Systems software package (SAS Institute, Cary, NC).

**Table 1:** Baseline characteristics of study participants (before after propensity score 1∶1 matching).

Variable	Before matching				After propensity matching			
	Aspirin users, (N = 2068)	Non-users, (N = 23272)	*p*-value	Standardized differences	Aspirin users, (N = 1884)	Non-users, (N = 1884)	*p*-value	Standardized differences
Age, year	61.7±13.0	48.5±15.7	**<0.001**	1.269	61.0±13.0	61.5±13.6	0.180	0.046
Male gender, n (%)	1213 (58.7)	11207 (48.2)	**<0.001**	0.212	1091 (57.9)	1048 (55.6)	0.164	0.046
Diabetes, n (%)	895 (43.3)	3606 (15.5)	**<0.001**	0.641	769 (40.8)	784 (41.6)	0.630	0.016
Hypertension, n (%)	1369 (66.2)	6285 (27.0)	**<0.001**	0.855	1203 (63.9)	1244 (66.0)	0.137	0.044
BMI, kg/m^2^	25.3±5.6	25.3±6.1	0.881	0.006	25.4±5.6	25.4±5.8	0.116	0.001
Previous CVD, n (%)	718 (34.7)	1082 (4.6)	**<0.001**	0.818	621 (33.0)	579 (30.7)	0.068	0.049
Previous PUD, n (%)	54 (2.6)	1089 (4.7)	**<0.001**	0.112	53 (2.8)	56 (3.0)	0.847	0.012
eGFR, ml/min/1.73 m^2^	42.3±28.1	69.8±29.3	**<0.001**	1.283	43.2±28.7	43.7±26.4	0.544	0.025
Proteinuria, n (%)	1268 (61.3)	10955 (47.1)	**<0.001**	0.288	1143 (60.7)	1185 (62.9)	0.261	0.045
Laboratory								
Hemoglobin, g/dl	12.3±2.6	13.2±2.2	**<0.001**	0.282	12.3±2.6	12.2±2.7	0.640	0.015
White blood cells, ×10^3^/µl	8.9±3.9	8.0±4.5	**<0.001**	0.267	8.8±3.8	8.8±4.1	0.891	0.008
Platelet, ×10^3^/µl	246.1±84.8	256.1±80.0	**<0.001**	0.164	246.6±85.0	252.5±102.4	0.052	0.080
Albumin, g/dl	3.8±0.7	4.2±0.6	**<0.001**	0.250	3.8±0.7	3.8±0.7	0.981	0.005
Cholesterol, mg/dl	193.2±60.3	188.3±47.3	**0.004**	0.112	193.1±60.3	192.4±59.1	0.854	0.017
Triglycerides, mg/dl	169.6±126.1	146.1±117.6	**<0.001**	0.263	168.9±127.5	173.9±142.4	0.347	0.049
HDL-cholesterol, mg/dl	44.6±13.7	50.9±14.7	**<0.001**	0.573	44.9±13.9	45.0±14.8	0.573	0.009
Calcium, mg/dl	8.9±0.8	9.1±0.7	**<0.001**	0.106	8.9±0.9	8.9±0.8	0.615	0.014
Phosphorus, mg/dl	3.9±1.2	3.7±1.6	**<0.001**	0.120	3.9±1.2	4.0±4.5	0.199	0.036
Uric acid, mg/dl	6.6±2.3	5.5±2.0	**<0.001**	0.478	6.6±2.3	6.5±2.4	0.638	0.037
Medications								
RAAS blockers, n (%)	1443 (69.8)	4973 (21.4)	**<0.001**	1.112	54 (2.9)	64 (3.4)	0.399	0.029
Statin, n (%)	813 (39.3)	2563 (11.0)	**<0.001**	0.690	10 (0.5)	21 (1.1)	0.072	0.067
Beta-blockers, n (%)	1205 (58.3)	3561 (15.3)	**<0.001**	0.996	37 (2.0)	39 (2.1)	0.907	0.020
CCB, n (%)	1275 (61.7)	4385 (18.8)	**<0.001**	0.973	44 (2.3)	55 (2.9)	0.315	0.038
Diuretics, n (%)	1250 (60.4)	4243 (18.2)	**<0.001**	0.958	65 (3.5)	74 (3.9)	0.488	0.021
Warfarin, n (%)	160 (7.7)	370 (1.6)	**<0.001**	0.293	2 (0.1)	0 (0)	0.500	0.045
Clopidogrel, n (%)	287 (13.9)	1084 (4.7)	**<0.001**	0.321	1 (0.1)	6 (0.3)	0.125	0.045
Cilostazol, n (%)	626 (30.3)	662 (2.8)	**<0.001**	0.797	3 (0.2)	2 (0.1)	>0.999	0.026

BMI: body mass index;

CVD: cardiovascular disease;

PUD: peptic ulcer disease;

eGFR: estimated glomerular filtration rate;

HDL: high-density lipoprotein;

RAAS: renin-angiotensin-aldosterone system;

CCB: calcium channel blocker.

*Note:* Conversion factors for units were as follows: hemoglobin in g/dl to g/l, ×10; white blood cell in ×10^3^/µl to ×10^9^/l, equal; platelets in ×10^3^/µl to ×10^9^/l, equal; albumin in mg/dl to g/l,×10; cholesterol in mg/dl to mmol/l,×0.02586; triglycerides in mg/dl to mmol/l,×0.01129; HDL-cholesterol in mg/dl to mmol/l,×0.02586; calcium in mg/dl to mmol/l, ×0.2495; phosphorus in mg/dl to mmol/l, ×0.3229; uric acid in mg/dl to µmol/l, ×59.48.

The continuous variable data are reported as the mean ± SD unless otherwise specified. The categorical data are expressed as the absolute values and percentages. Continuous variables were compared by Student’s t-tests, and categorical variables were compared by χ^2^ or Fisher’s exact tests, as appropriate before PS matching. Following the descriptive statistics, cumulative incidences of primary and secondary end points were estimated by the Kaplan-Meier method, and differences between groups were assessed with the log-rank test. A Cox proportional hazard model was used to determine the independent risk factors for incidence of atherosclerotic CVD and to estimate hazard ratio (HR) of aspirin use along with 95% confidence intervals (CIs) for incidence of doubling of serum creatinine and ESRD. The adjustment variables included age (10-year increase), diabetic status, hypertension, history of CVD, proteinuria, baseline eGFR, hemoglobin level (<10 g/dl), albumin level (<3.5 g/dl), and cholesterol as well as the use of RAAS blockers, statins, beta-blockers, CCBs, diuretics, warfarin, clopidogrel and cilostazol. A Cox proportional hazard model stratified by matched pairs was also used for the matched cohort. All analyses except PS matching were performed with SPSS version 15.0 software (SPSS, Inc., Chicago, IL). Statistical significance was defined as *P*<0.05.

## Results

### Study population and baseline characteristics

A total of 34024 patients who presented with renal complications were screened for inclusion in this study, and a total of 25340 patients fulfilled the inclusion criteria. The cohort with aspirin use included patients who were already taking aspirin at the time they enrolled in the study. Aspirin was prescribed for 8.2% (2068 of 25340) of the total number of patients. The characteristics of the study population are summarized in Table 1. Patients who were prescribed aspirin were more likely than non-users to be older and male. These patients were also more likely to have diabetes; hypertension; previous CVD; proteinuria; low baseline kidney function; low levels of hemoglobin, platelets, albumin, HDL cholesterol, and calcium; and high levels of total cholesterol, triglycerides, phosphorus, uric acid, and white blood cells. In addition, these patients were less likely to have peptic ulcer disease.

BMI and medication status (e.g., RAAS blockers, statins, beta-blockers, CCB, diuretics, warfarin, clopidogrel, and cilostazol) did not vary significantly between aspirin users and non-users. Using PS matching, 1884 aspirin users were successfully matched to non-users (Figure 1). After PS matching, there were no statistically significant clinical differences between the aspirin users and the non-users (Table 1).

### Risk factors for atherosclerotic CVD

**Figure 2:**
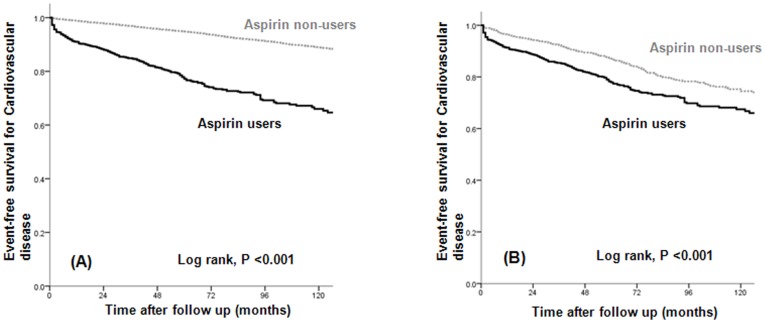
The event-free survival of patients with atherosclerotic cardiovascular disease according to treatment group (A) before and (B) after PS matching. Atherosclerotic cardiovascular disease occurred more frequently in aspirin users versus non-users.

Atherosclerotic CVD occurred significantly more often in aspirin users before and after PS matching (*P*<0.001; Figure 2). We used Cox proportional hazard analyses to evaluate the possible risk factors for the development of atherosclerotic CVD. In a multivariate Cox proportional analysis adjusted for aspirin use, age (10-year increase), baseline eGFR, presence of proteinuria, previous CVD, diabetes, hypertension, hemoglobin level <10 g/dl, albumin level <3.5 g/dl, cholesterol level, and use of medications (RAAS blockers, statins, beta-blockers, CCBs, diuretics, warfarin, clopidogrel, and cilostazol), the risk of atherosclerotic CVD was significantly higher in aspirin users than in non-users in the unmatched cohort (HR, 2.577; 95% CI, 2.238–2.967; *P*<0.001; Table 2). In the matched cohort, the use of aspirin was also associated with a higher incidence of atherosclerotic CVD (HR, 2.259; 95% CI, 1.880–2.714; *P*<0.001; Table 2).

**Table 2:** Multivariate Cox proportional analyses for atherosclerotic cardiovascular disease.

	Unmatched cohort		Matched cohort	
	HR (95% CI)	*p*-value	HR (95% CI)	*p*-value
Aspirin	2.577 (2.238–2.967)	**<0.001**	2.259 (1.880–2.714)	**<0.001**
Age, 10-year increase	1.383 (1.314–1.456)	**<0.001**	1.171 (1.085–1.264)	**<0.001**
Baseline eGFR	0.995 (0.993–0.998)	**0.001**	0.995 (0.991–0.999)	**0.013**
Proteinuria	1.032 (0.903–1.178)	0.645	0.929 (0.763–1.131)	0.463
Previous CVD	3.282 (2.870–3.754)	**<0.001**	2.866 (2.393–3.431)	**<0.001**
Diabetes	1.731 (1.525–1.964)	**<0.001**	1.592 (1.334–1.899)	**<0.001**
Hypertension	2.291 (1.968–2.666)	**<0.001**	1.493 (1.203–1.851)	**<0.001**
Hemoglobin<10 g/dl	0.728 (0.569–0.932)	**0.012**	0.657 (0.481–0.897)	**0.008**
Albumin <3.5 g/dl	1.026 (0.845–1.247)	0.794	0.935 (0.722–1.210)	0.608
Cholesterol	1.002 (1.001–1.003)	**<0.001**	1.001 (1.000–1.003)	0.068
’RAAS blockers	0.685 (0.495–0.947)	**0.022**	0.802 (0.466–1.380)	0.426
Statin	1.021 (0.613–1.702)	0.935	0.320 (0.045–2.298)	0.257
Beta-blockers	0.954 (0.695–1.310)	0.772	0.998 (0.582–1.710)	0.994
CCB	1.146 (0.883–1.488)	0.306	0.837 (0.482–1.454)	0.528
Diuretics	0.676 (0.488–0.936)	**0.018**	0.649 (0.351–1.198)	0.167
Warfarin	1.079 (0.473–2.458)	0.857	3.820 (0.495–29.509)	0.199
Clopidogrel	3.049 (1.438–6.465)	**0.004**	3.384 (0.826–13.863)	0.090
Cilostazol	2.159 (1.237–3.767)	**0.007**	1.294 (0.178–9.405)	0.799

Abbreviations: HR: hazards ratio;

eGFR: estimated glomerular filtration rate;

CVD: cardiovascular disease;

RAAS: renin-angiotensin-aldosterone system;

CCB: calcium channel blocker.

### Association between aspirin use and diabetes and between aspirin use and beta-blockers for cardiovascular events

**Figure 3:**
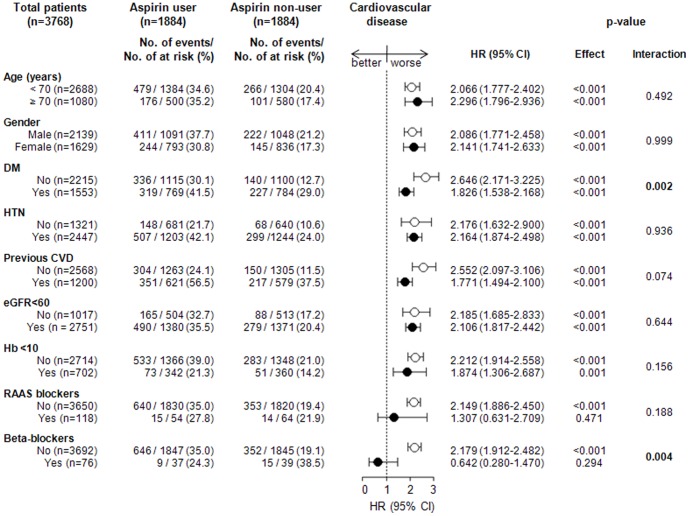
Association of aspirin use and cardiovascular disease in subgroups of the matched cohort.

Of the 3768 patients included in the study, 1553 (41.2%) were diagnosed with diabetes. A total of 319 of 769 (41.5%) PS-matched diabetic patients used aspirin, while 336 of 1115 (30.1%) PS-matched non-diabetic patients used aspirin. The HRs (95% CIs) for CVD among those with and without diabetes were 1.826 (1.538–2.168, *P*<0.001) and 2.646 (2.171–3.225, *P*<0.001), respectively (*P* for interaction, 0.002; Figure 3). A total of 646 of 1847 (35.0%) matched patients treated with beta-blockers used aspirin, while 9 of 37 (24.3%) matched patients treated without beta-blockers used aspirin. The HRs (95% CIs) for CVD among those treated with and without beta-blockers were 2.179 (1.912–2.482, *P*<0.001) and 0.642 (0.280–1.470, *P* = 0.294), respectively (*P* for interaction, 0.004; Figure 3). The associations between aspirin use and CVD were generally homogeneous across the other patient subgroups (Figure 3).

### Risk of bleeding associated with aspirin use

**Figure 4:**
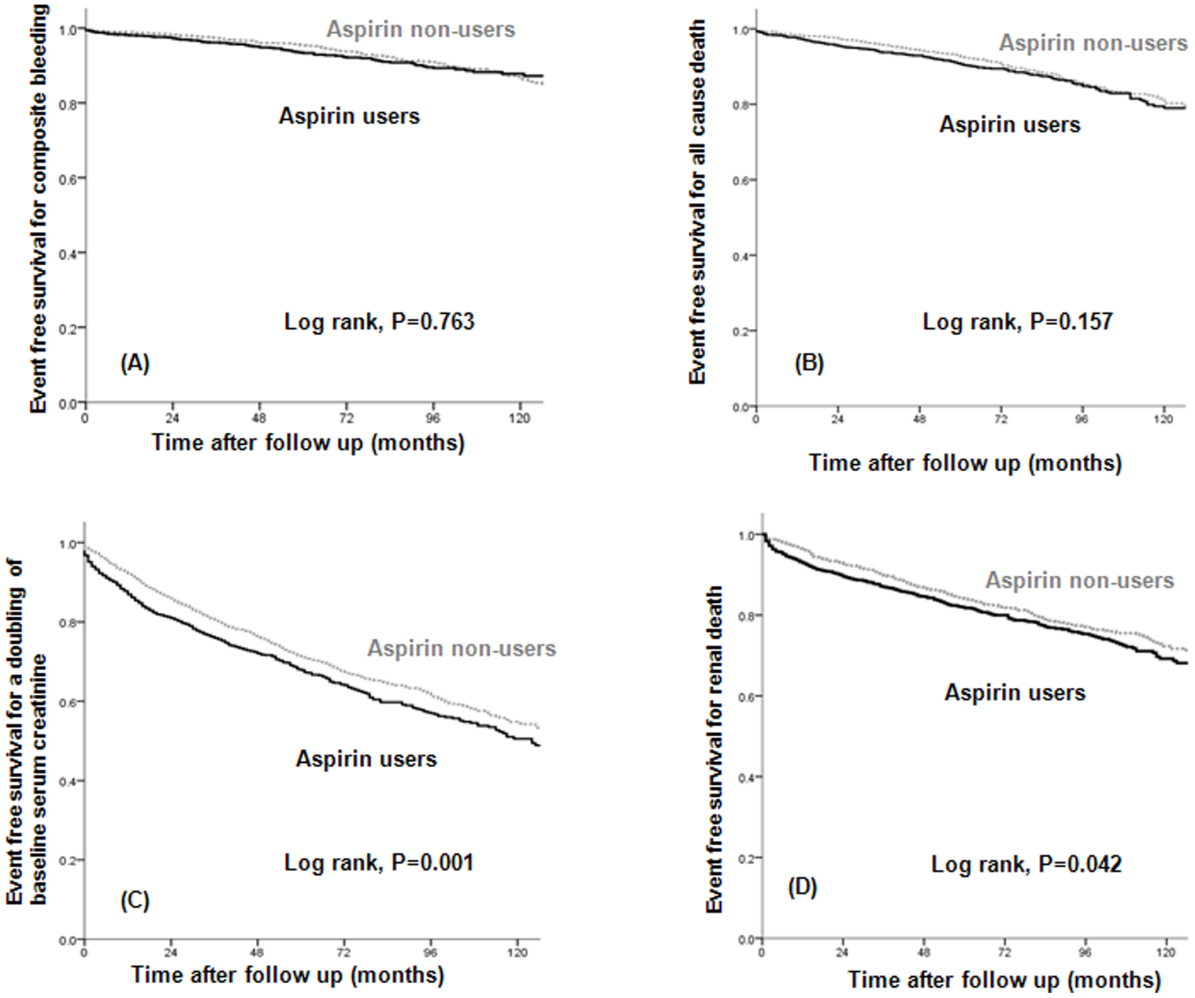
The event-free survival for (A) composite bleeding, (B) all-cause death, (C) a doubling of baseline serum creatinine, and (D) renal death according to treatment group in the matched cohort. There were no significant differences in the incidence of composite bleeding and the cumulative overall survival rate between aspirin users and non-users. Aspirin users showed a higher incidence of doubling of baseline serum creatinine and an increased renal death rate compared with non-users.

The composite of gastrointestinal bleeding, hemorrhagic stroke, and hemoptysis was not significantly different between the aspirin users and non-users in the matched cohort (*P* = 0.763; Figure 4A).

### Other secondary endpoints: survival and renal outcome

We also analyzed the cumulative survival rate between patients who were prescribed aspirin and those who were not. There were no significant differences in the cumulative overall survival rate between aspirin users and non-users in the matched cohort (*P* = 0.157; Figure 4(B)). The event-free survival, the doubling of serum creatinine (*P* = 0.001; Figure 4C), and renal death (*P* = 0.042; Figure 4D) were significantly lower in the aspirin users than in the non-users. We performed a multivariate Cox proportional analysis that was adjusted for aspirin use, age (10–year increase), baseline eGFR, presence of proteinuria, previous CVD, diabetes, hypertension, hemoglobin level (<10 g/dl), albumin level (<3.5 g/dl), and the use of medications (e.g., RAAS blockers, statins, beta-blockers, CCBs, diuretics, warfarin, clopidogrel, and cilostazol). Even after adjustment, the risks of serum creatinine doubling and renal death were significantly higher in aspirin users than in non-users in the unmatched cohort (HR, 1.390; 95% CI, 1.255–1.540; *P*<0.001 for doubling of serum creatinine and HR, 1.467; CI, 1.279–1.683; *P*<0.001 for renal death; Table 3) and matched cohort (HR, 1.325; 95% CI, 1.160–1.513; *P*<0.001 for doubling of serum creatinine and HR, 1.310; CI, 1.096–1.566; *P* = 0.003 for renal death; Table 3).

**Table 3:** Multivariate Cox proportional analyses for a doubling of serum creatinine and ESRD^a^.

	Unmatched cohort		Matched cohort	
	HR (95% CI)	p-value	HR (95% CI)	p-value
SCr x 2b	1.390 (1.255–1.540)	**<0.001**	1.325 (1.160–1.513)	**<0.001**
ESRD	1.467 (1.279–1.683)	**<0.001**	1.310 (1.096–1.566)	**0.003**

Abbreviations: HR: hazards ratio;

CI: confidence interval;

SCr: serum creatinine;

ESRD: end-stage renal disease.

a adjusted for aspirin treatment, age (10 year increase), baseline eGFR, proteinuria, previous CVD, diabetes, hypertension, hemoglobin (<10 g/dl), and albumin (<3.5 g/dl) levels and use of medications (RAAS blockers, statin, beta-blockers, CCB, diuretics, warfarin, clopidogrel, cilostazol).

b SCr x 2: a doubling of the baseline serum creatinine concentration.

## Discussion

In the present study, we evaluated the role of low-dose aspirin in the prevention of atherosclerotic CVD in patients with CKD. Using PS matching, we found that patients with CKD who were on a regimen of low-dose aspirin were significantly more likely to develop atherosclerotic CVD than those who did not use aspirin. Even after the adjustment for underlying CVD, the use of aspirin remained an independent and significant predictor of CVD. In the subgroup analyses, almost all subgroups were consistently associated with an increased risk of CVD. Although there were no significant differences in all-cause mortality or composite bleeding events between the aspirin users and non-users, doubling of serum creatinine and renal death occurred more frequently in the aspirin users. These findings are interesting in light of the use of low-dose aspirin for protection against CVD in this population.

The burden of CVD among patients with CKD is substantial. The increased risk of CVD and mortality in patients with CKD has stimulated interest in identifying effective methods for reducing the risk of CVD. Although the effects of aspirin in individuals with CKD are uncertain, several studies support the beneficial effect of aspirin in this population. Sciahbasi et al [17] and McCullough et al [18] reported that aspirin use was associated with reduced odds of admission with ST elevation MI and reduced in-hospital mortality after MI. In a post hoc subgroup analysis of a randomized controlled trial, Jardine et al [19] also reported that aspirin therapy led to greater absolute reduction in major CVD and greater mortality in hypertensive patients with CKD than in patients with normal kidney function. These authors explain that the increased benefit lies partially in the high baseline risk of these patients, which translates a similar proportional benefit into a greater absolute benefit.

A recent meta-analysis by Palmer et al [20] evaluated the effects of aspirin and clopidogrel in 9 trials (all post hoc subgroup analyses for CKD) involving 9969 patients who had acute ACS or who were undergoing percutaneous coronary intervention (PCI) and in 31 trials involving 11,701 patients with stable or no CVD. These authors concluded that the use of antiplatelet agents in patients with CKD had little or no effect on all-cause or cardiovascular mortality or on myocardial infarction. However, these authors acknowledged the limitations of low-quality or very low-quality evidence (i.e., significant heterogeneity in the study populations, considerable variation among the studies, and variations in the post hoc subgroup analyses of the trials).There are several potential mechanisms that may explain the poor response to treatment with antiplatelet agents in patients with CKD, including increased platelet activation [11], high residual platelet reactivity [11], [21], altered pharmacokinetic effects of uremia on drug transport and non-renal metabolism [22], [23], and elevated Von Willebrand antigen levels [24], [25] in these patients. In addition, patients with CKD not only have thrombotic predisposition [26] but also, paradoxically, have bleeding diathesis [27] due to the underlying complex hemostatic disorder found in individuals with progressive kidney failure. Uremia is associated with prolongation of bleeding time as well as abnormal platelet aggregation and adhesion due to intrinsic and extrinsic factors [28].

In the subgroup analysis, all subgroups were associated with an increased risk for CVD, except the subjects who were being treated with RAAS blockers and beta-blockers. We evaluated the association between aspirin use (in addition to diabetes and beta-blockers) and other factors for CVD. Aspirin use is associated with a lower HR for CVD in diabetic patients compared to non-diabetic patients. This finding may support the recommendation of low-dose aspirin for secondary prevention of CVD in diabetic patients. In addition, aspirin use is also associated with a lower HR for CVD in individuals who use beta-blockers compared to those who do not. This effect may also be due to the protective role of beta-blockers against CVD.

The question of whether the use of aspirin by patients with CKD increases the risk for bleeding is controversial. The first United Kingdom Heart and Renal Protection (UK-HARP) trial [29] and the Dialysis Outcomes and Practice Patterns Study (DOPPS) [30] showed no increased risk of major bleeding, gastrointestinal bleeding in those who were taking an aspirin dose of 100 mg/day, respectively. However, in the meta-analysis by Palmer et al [20], there was an increase in major and minor bleeding events with the use of antiplatelet agents in patients with CKD and ACS who required PCI. These findings were generated using low-quality evidence, with considerable variation in trial duration, heterogeneity in the definitions and assessment of bleeding outcomes, and reliance on subgroup data from major trials. The incidence of a bleeding event in our study may be lower than the incidence in these other studies because we defined the bleeding risk as a composite bleeding event that includes hemorrhagic stroke, gastrointestinal bleeding, and hemoptysis and does not include minor bleeding such as epistaxis, ecchymosis, or bruising. However, we believe that our criteria are also suitable for defining a clinically significant bleeding event. Additional well-designed and controlled studies are needed to confirm the association between the use of antiplatelet agents and the risk of bleeding in patients with CKD.

We assessed the effect of low-dose aspirin on renal outcome in patients with CKD. The doubling of serum creatinine and the occurrence of ESRD that requires renal replacement therapy were significantly associated with low-dose aspirin use in patients with CKD. Unlike our study, The UK-HARP-1 trial [29] showed that low-dose aspirin use in patients with CKD was not associated with progression of CKD (as measured by a decrease in renal function, >20% increase in creatinine, and the initiation of dialysis). Subsequently, in the subgroup analysis of a randomized controlled trial, Jardine et al [19] reported that aspirin use did not affect renal function in the overall study population, but data were scarce regarding the effects of low-dose aspirin on the renal outcome in patients within any eGFR category. However, because the study of Jardine et al is a post hoc subgroup analysis of hypertension optimal treatment (HOT), which mainly included diastolic hypertensive patients, only 2.9% of the study population had an eGFR<45 ml/min/1.73 m^2^, in contrast to 53.1% in our study.

Although our study showed that the use of aspirin was associated with a higher risk for CVD even after the adjustment for any underlying CVD, there were some limitations. First, the participants in this study were from a single center and single ethnic groups. There need to be a further investigation about the effect of low-dose aspirin on atherosclerotic cardiovascular disease among patients with chronic kidney disease in multi-center, other ethnic and racial groups. Second, this study was an observational study rather than a controlled interventional trial. Thus, some important baseline covariates were not distributed equally. To address this issue, we used PS matching to minimize the differences in baseline characteristics. Despite these efforts, differences in unmeasured characteristics could have led to biased results. Third, we could not evaluate whether non-use of aspirin could lead to beneficial effects on CVD development in these patients because our study was not an interventional trial.

Despite these limitations, our study is the only large observational cohort study with PS matching that provides evidence that the use of low-dose aspirin is associated with a higher risk for atherosclerotic CVD in patients with CKD.

In conclusion, although the role of low-dose aspirin in the development of atherosclerotic CVD in patients with CKD is debatable, our results show that the use of low-dose aspirin in patients with CKD has potentially harmful effects, as it increases the risk for CVD and renal progression. Further randomized clinical trials are warranted to confirm the effect of low-dose aspirin therapy on the development of CVD in these patients.

## References

[1] Coresh J, Selvin E, Stevens LA, Manzi J, Kusek JW, et al (2007) Prevalence of chronic kidney disease in the United States. JAMA 298: 2038–2047. 17986697 10.1001/jama.298.17.2038

[2] Ninomiya T, Perkovic V, Turnbull F, Neal B, Barzi F, et al (2013) Blood pressure lowering and major cardiovascular events in people with and without chronic kidney disease: meta-analysis of randomised controlled trials. BMJ 347: f5680. 24092942 10.1136/bmj.f5680 PMC3789583

[3] Mann JF, Gerstein HC, Pogue J, Bosch J, Yusuf S (2001) Renal insufficiency as a predictor of cardiovascular outcomes and the impact of ramipril: the HOPE randomized trial. Ann Intern Med 134: 629–636. 11304102 10.7326/0003-4819-134-8-200104170-00007

[4] Palmer SC, Craig JC, Navaneethan SD, Tonelli M, Pellegrini F, et al (2012) Benefits and harms of statin therapy for persons with chronic kidney disease: a systematic review and meta-analysis. Ann Intern Med 157: 263–275. 22910937 10.7326/0003-4819-157-4-201208210-00007 PMC3955032

[5] Baigent C, Blackwell L, Collins R, Emberson J, Godwin J, et al (2009) Aspirin in the primary and secondary prevention of vascular disease: collaborative meta-analysis of individual participant data from randomised trials. Lancet 373: 1849–1860. 19482214 10.1016/S0140-6736(09)60503-1 PMC2715005

[6] Pignone M, Alberts MJ, Colwell JA, Cushman M, Inzucchi SE, et al (2010) Aspirin for primary prevention of cardiovascular events in people with diabetes: a position statement of the American Diabetes Association, a scientific statement of the American Heart Association, and an expert consensus document of the American College of Cardiology Foundation. Circulation 121: 2694–2701. 20508178 10.1161/CIR.0b013e3181e3b133

[7] U.S. Preventive Services Task Force (2009) Aspirin for the prevention of cardiovascular disease: U.S. Preventive Services Task Force recommendation statement. Ann Intern Med 150: 396–404. 19293072 10.7326/0003-4819-150-6-200903170-00008

[8] Oberg BP, McMenamin E, Lucas FL, McMonagle E, Morrow J, et al (2004) Increased prevalence of oxidant stress and inflammation in patients with moderate to severe chronic kidney disease. Kidney Int 65: 1009–1016. 14871421 10.1111/j.1523-1755.2004.00465.x

[9] Boccardo P, Remuzzi G, Galbusera M (2004) Platelet dysfunction in renal failure. Semin Thromb Hemost 30: 579–589. 15497100 10.1055/s-2004-835678

[10] Adeseun GA, Xie D, Wang X, Joffe MM, Mohler ER 3rd, et al (2012) Carotid plaque, carotid intima-media thickness, and coronary calcification equally discriminate prevalent cardiovascular disease in kidney disease. Am J Nephrol 36: 342–347. 23107930 10.1159/000342794 PMC3538165

[11] Gremmel T, Muller M, Steiner S, Seidinger D, Koppensteiner R, et al (2013) Chronic kidney disease is associated with increased platelet activation and poor response to antiplatelet therapy. Nephrol Dial Transplant 28: 2116–2122. 23729489 10.1093/ndt/gft103

[12] Debella YT, Giduma HD, Light RP, Agarwal R (2011) Chronic kidney disease as a coronary disease equivalent–a comparison with diabetes over a decade. Clin J Am Soc Nephrol 6: 1385–1392. 21393492 10.2215/CJN.10271110 PMC3109936

[13] Briasoulis A, Bakris GL (2013) Chronic kidney disease as a coronary artery disease risk equivalent. Curr Cardiol Rep 15: 340. 23338722 10.1007/s11886-012-0340-4

[14] Charytan D, Kuntz RE (2006) The exclusion of patients with chronic kidney disease from clinical trials in coronary artery disease. Kidney Int 70: 2021–2030. 17051142 10.1038/sj.ki.5001934 PMC2950017

[15] Weigert AL, Schafer AI (1998) Uremic bleeding: pathogenesis and therapy. Am J Med Sci 316: 94–104. 9704663 10.1097/00000441-199808000-00005

[16] National Kidney Foundation (2002) K/DOQI clinical practice guidelines for chronic kidney disease: evaluation, classification, and stratification. Am J Kidney Dis 39: S1–266. 11904577

[17] Sciahbasi A, Arcieri R, Quarto M, Pendenza G, Lanzillo C, et al (2010) Impact of chronic aspirin and statin therapy on presentation of patients with acute myocardial infarction and impaired renal function. Prev Cardiol 13: 18–22. 20021622 10.1111/j.1751-7141.2009.00050.x

[18] McCullough PA, Sandberg KR, Borzak S, Hudson MP, Garg M, et al (2002) Benefits of aspirin and beta-blockade after myocardial infarction in patients with chronic kidney disease. Am Heart J 144: 226–232. 12177638 10.1067/mhj.2002.125513

[19] Jardine MJ, Ninomiya T, Perkovic V, Cass A, Turnbull F, et al (2010) Aspirin is beneficial in hypertensive patients with chronic kidney disease: a post-hoc subgroup analysis of a randomized controlled trial. J Am Coll Cardiol 56: 956–965. 20828648 10.1016/j.jacc.2010.02.068

[20] Palmer SC, Di Micco L, Razavian M, Craig JC, Perkovic V, et al (2012) Effects of antiplatelet therapy on mortality and cardiovascular and bleeding outcomes in persons with chronic kidney disease: a systematic review and meta-analysis. Ann Intern Med 156: 445–459. 22431677 10.7326/0003-4819-156-6-201203200-00007

[21] Angiolillo DJ, Bernardo E, Capodanno D, Vivas D, Sabate M, et al (2010) Impact of chronic kidney disease on platelet function profiles in diabetes mellitus patients with coronary artery disease taking dual antiplatelet therapy. J Am Coll Cardiol 55: 1139–1146. 20223369 10.1016/j.jacc.2009.10.043

[22] Dreisbach AW (2009) The influence of chronic renal failure on drug metabolism and transport. Clin Pharmacol Ther 86: 553–556. 19776735 10.1038/clpt.2009.163

[23] Nolin TD, Frye RF, Le P, Sadr H, Naud J, et al (2009) ESRD impairs nonrenal clearance of fexofenadine but not midazolam. J Am Soc Nephrol 20: 2269–2276. 19696225 10.1681/ASN.2009010082 PMC2754095

[24] Shen L, Lu G, Dong N, Jiang L, Ma Z, et al (2012) Von Willebrand factor, ADAMTS13 activity, TNF-alpha and their relationships in patients with chronic kidney disease. Exp Ther Med 3: 530–534. 22969924 10.3892/etm.2011.432 PMC3438530

[25] Spiel AO, Gilbert JC, Jilma B (2008) von Willebrand factor in cardiovascular disease: focus on acute coronary syndromes. Circulation 117: 1449–1459. 18347221 10.1161/CIRCULATIONAHA.107.722827

[26] Wattanakit K, Cushman M, Stehman-Breen C, Heckbert SR, Folsom AR (2008) Chronic kidney disease increases risk for venous thromboembolism. J Am Soc Nephrol 19: 135–140. 18032796 10.1681/ASN.2007030308 PMC2391038

[27] Mezzano D, Tagle R, Panes O, Perez M, Downey P, et al (1996) Hemostatic disorder of uremia: the platelet defect, main determinant of the prolonged bleeding time, is correlated with indices of activation of coagulation and fibrinolysis. Thromb Haemost 76: 312–321. 8883263

[28] Washam JB, Adams GL (2008) Risks and benefits of antiplatelet therapy in uremic patients. Adv Chronic Kidney Dis 15: 370–377. 18805383 10.1053/j.ackd.2008.07.006

[29] Baigent C, Landray M, Leaper C, Altmann P, Armitage J, et al (2005) First United Kingdom Heart and Renal Protection (UK-HARP-I) study: biochemical efficacy and safety of simvastatin and safety of low-dose aspirin in chronic kidney disease. Am J Kidney Dis 45: 473–484. 15754269 10.1053/j.ajkd.2004.11.015

[30] Ethier J, Bragg-Gresham JL, Piera L, Akizawa T, Asano Y, et al (2007) Aspirin prescription and outcomes in hemodialysis patients: the Dialysis Outcomes and Practice Patterns Study (DOPPS). Am J Kidney Dis 50: 602–611. 17900460 10.1053/j.ajkd.2007.07.007

